# Acknowledgement to reviewers 2024

**DOI:** 10.1530/EOR-10-A1

**Published:** 2025-01-03

The Editor-in-Chief would like to thank the following reviewers for their valued support in 2024:

K Abdelkader

A Acker

M C de Andres

P Andrés-Cano

J-N Argenson

B Atilla

N Attal

D Back

A Badke

J Bago

G Balato

Z Balogh

R Barco

P Bas

J-C Bel

D N Bhatia

K Bilsel

O Birsel

C Biz

M Borroni

P Bouché

S Boudabbous

D Braconi

M Brix

E Brzoska

L Campanacci

F Canavese

S Cannon

J L Cebrian-Carretero

A Chandrinou

H Chase

M Clauss

W Colyn

C Combescure

P Consigliere

J Cordero-Ampuero

C Coster

A Covaro

P Crespo lastras

D Dallari

R D'Apolito

R De Steiger

D Dejour

A Del Arco

M Demirhan

E Duarte

D Eastwood

P J Erasmus

J Erickson

C Evans

G U Exner

X Flecher

T Freude

C Frolich Frandsen

W Fu

J Gallo

E García Cimbrelo

A García de Frutos

E García-Rey

P Giannoudis

S Gill

J Girard

M E Gomes

A Gonzalez

R Gonzalez

G Gonzalez-Morán

S B Goodman

T Gottlieb

E Guerado

K Günther

T Hamilton

D Hannouche

N Harris

P Hernandez-Esteban

P Hernigou

F Hinterwimmer

A Höch

K Huang

G Hussain

M A Imam

F Imhoff

A Imhoff

A Isart

E Itoi

V Jaecker

K Jeno

S Johnson

M Jokiel

A A Kaissi

N Kanakaris

J C Katthagen

M Kicinski

P Kjaersgaard-Andersen

F Klenke

H Klobučar

S Kopf

H Kuo

A Kwapisz

P Laboudie

S Lambert

D Langton

R LaPrade

H Laprus

E Lebel

B Lecumberri

W Lehmann

A Leithner

O Levy

A Lübbeke

G A Lucidi

S J MacDessi

G Macheras

E Mah

F Maselli

E V McCloskey

A Mediero

S Meller

J Menetrey

P Menon

H Migaud

S Miklós

H Miozzari

M Mohaddes

F Molina

J R Murray

M Nabergoj

E Naredo

A Nedopil

S Newman

V Nikolaou

D C Noriega

P Norwa

A Nuessler

N Ozeki

P Paladini

P Papagelopoulos

R Papalia

A Peiro

H Pereira

C Perisano

C Perka

F Perut

M Pes

D Petek

C Plaass

I-A Popescu

N Reina

C Rivière

S A Rodeo

O Rolfson

P Ruggieri

M Ruiz Ban

P Sadoghi

L Saldaña

V Salini

G Saló

N Santori

W Schreurs

M Schultz Larsen

A Seitz

A Senkoylu

T Seow Hui

W Shrader

K Siebenrock

C Simon Perez

G Skaliczki

E Slette

G Solarino

M Soylemez

N Spranger

D Suva

Y Takegami

M Tegern

S D A L Tobing

F Tomé-Bermejo

A Trampuz

P Tscholl

M Ubierna

M Ulloa

B Unver

G Vadala

P Vadillo

M Van Nuffel

R Verdonk

G Vilà-Canet

J F Vilchez-Cavazos

M Walther

X-Q Wang

D Warwick

P Weber

J Windolf

T Winkler

P Winnock de Grave

J Wong-Chung

J Xie

G Zanoli

G Zatti

